# LGBTIQ+ Specialty Care in USA: The Continuing Need for Specialty Clinics and Resources.

**DOI:** 10.1192/j.eurpsy.2025.88

**Published:** 2025-08-26

**Authors:** A. Ahuja, LGBTQ

**Affiliations:** Los Angeles LGBT Center, Los Angeles, United States

## Abstract

**Abstract:**

This presentation will serve as an overview of LGBTQ+ Clinical Care in the United States and throughout the world. Using the specific example of the Los Angeles LGBT Center, Dr. Ahuja will discuss the need for an LGBTQ+-specific mental health clinic. The reasons for having specialized care are numerous, but boil down to several significant areas.

First, the LGBTQ+ community suffers from large health disparities. This often results from lack of specialized knowledge by providers and health advocates. An LGBTQ+ specific center allows for experts to exchgange and enhance information, incerased research into this population which results in better standars of care, and a chance to educate others regarding LGBTQ+ care. They can serve as a model for other clinics or any other health settings where practitioners can implement LGBTQ+-specific practices that work for them and their patients and on a scale that makes sense.

Second, there is much evidence of discrimination by healthcare providers and insurance companies regarding the LGBTQ+ community, particularly when it comes to the Transgender population. This can include rude behavior by providers, missed screenings due to not paying attention to patient anatomy or LGBTQ+ status, misgendering, harrasment, and lack of insurance coverage for procedures. An LGBTQ+ center allows for patients to have healthcare advocates who can fight for approval and ensures that patients are not harrassed for being LGBTQ+ which results in many patients forgoing necessary care. Further, screenings and patient education regarding things like Pre-Exposure Prophylaxis and Hormone Therapy are much improved as the center and its providers have the knowledge and mission to carry out large-scale educaton efforts.

Finally, an LGBTQ+ center like the Los Angeles LGBT Center inevitably becomes a community hub. These centers function to not just provide medical care but also to advocate for LGBTQ+ issues, coordinate with other centers to manage population disease outbreaks, fight misinformation and disinformation with accurate research, and provide many ancillary services including housing and social community spaces.

Select Sources

James, S.E., Herman, J.L., Durso, L.E., & Heng-Lehtinen, R. (2024). Early Insights: A Report of the 2022 U.S. Transgender Survey. National Center for Transgender Equality, Washington, DC.

Holt S, Ahuja A (2025). LGBTQ+ Intimate Partner Violence: A Guide for Mental Health Practitioners. Routledge.

**Disclosure of Interest:**

None Declared

